# Ongoing large measles outbreak with nosocomial transmission in Milan, northern Italy, March–August 2017

**DOI:** 10.2807/1560-7917.ES.2017.22.33.30596

**Published:** 2017-08-17

**Authors:** Antonella Amendola, Silvia Bianchi, Elena R Frati, Giulia Ciceri, Marino Faccini, Sabrina Senatore, Daniela Colzani, Anna Lamberti, Melissa Baggieri, Danilo Cereda, Maria Gramegna, Loredana Nicoletti, Fabio Magurano, Elisabetta Tanzi

**Affiliations:** 1 Department of Biomedical Sciences for Health, University of Milan, Milan, Italy; 2 Coordinated Research Center ‘EpiSoMI’, University of Milan, Milan, Italy; 3 Health Protection Agency, Metropolitan Area of Milan, Milan, Italy; 4 National Reference Laboratory for Measles and Rubella, Istituto Superiore di Sanità, Rome, Italy; 5 DG Salute, UO Governo della prevenzione e tutela sanitaria, Lombardy Region, Milan, Italy

**Keywords:** Measles, Surveillance, Outbreaks, Nosocomial transmission, Epidemiology, Phylogeny

## Abstract

A large measles outbreak has been ongoing in Milan and surrounding areas. From 1 March to 30 June 2017, 203 measles cases were laboratory-confirmed (108 sporadic cases and 95 related to 47 clusters). Phylogenetic analysis revealed the co-circulation of two different genotypes, D8 and B3. Both genotypes caused nosocomial clusters in two hospitals. The rapid analysis of epidemiological and phylogenetic data allowed effective surveillance and tracking of transmission pathways.

A large measles outbreak has been ongoing in Milan and surrounding areas, a densely populated area with nearly 4 million inhabitants. Rapid and active surveillance was set up by the Subnational Reference Laboratories (SRL) Milan, established as part of the measles and rubella surveillance network MoRoNet [1] in March 2017, with 303 investigated cases at the time of submission of this report. We present a detailed analysis of the period 1 March to 30 June 2017, with the aim to conduct a complete and rapid characterisation of wild-type measles virus (MV) strains circulating. 

## Confirmation and investigation of cases and clusters in Milan

From 1 March to 30 June 2017, 233 suspected cases of measles were investigated: there were 203 (87%) laboratory-confirmed cases (median age: 30 years; range: 2 months–77 years) and 30 (13%) were discarded. Overall 60% (n = 121) of the confirmed cases were individuals aged 15–39 years and 6% (n = 12) were ≤ 1 year of age; 88% (n = 179) were not vaccinated and 12% (n = 24) were vaccinated (six with two doses of measles-mumps-rubella (MMR) vaccine, 10 with one dose, and eight did not know the number of doses). According to the epidemiological regional database, 108 of 203 were sporadic cases and 95 were related to 47 clusters. Cases were classified as sporadic when an epidemiological link to other cases could not be established.

## Molecular surveillance for cases and clusters

The genotype of MV strains was successfully identified in 187 of 203 (92%) of the confirmed cases by sequencing the highly variable region of nucleoprotein (N) gene (N-450) [2]. Phylogenetic analysis revealed that the MV strains belonged to genotypes D8 and B3.

The most common genotype detected was genotype D8 (86%; 160/187 cases) which was related to 77 cases in 42 clusters and 83 sporadic cases. All of the D8 cases and clusters were autochthonous or from unknown source.

**Figure 1:**
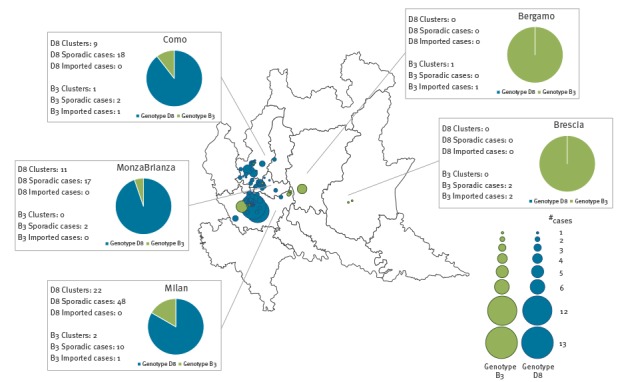
Geographical distribution of measles cases and clusters, with genotypes, Milan, 1 March–30 June 2017 (n = 187 cases)

**Figure 2:**
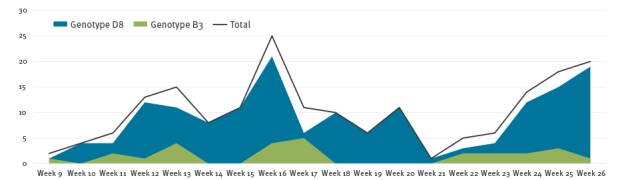
Temporal distribution of measles cases and genotypes detected, Milan, 1 March–30 June 2017 (n = 187)

In March 2017, the B3 genotype was detected in five imported cases: two sporadic cases and three cases which subsequently caused three import-related clusters. From April to end of June 2017, a further 14 autochthonous sporadic cases and one cluster were reported. The geographical and temporal distribution, respectively, of sporadic cases and clusters related to D8 and B3 genotypes are shown in Figure 1 and Figure 2.

### Measles virus genotype D8

Phylogenetic analysis showed that all the D8 sequences (n = 160) fell into the Osaka lineage (MVi/Osaka.JPN/29.15; similarity range: 99–100%). From March to May, D8 MV strains mainly caused clusters in work and family settings, which occurred principally in the north-eastern area of Milan. During the week starting on 27 March 2017, a serious family cluster affected three cases causing the death of one of them. The D8 MV strain was isolated from the biological samples of one of these cases.

**Figure 3:**
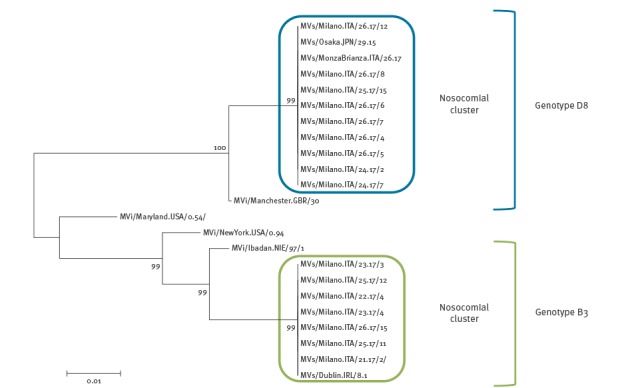
Neighbour-joining tree for nucleotide sequences of measles virus D8 and B3 variants causing nosocomial clusters in two hospitals, Milan, 1 March–30 June 2017 (n = 16)

In June 2017, the D8 Osaka variant spread through the city of Milan and caused a nosocomial cluster in a hospital. Based on epidemiological data, a link was established between 12 cases: eight healthcare workers (HCWs), three patients and one visitor. Phylogenetic analysis showed that the nine sequences obtained were 100% identical. The match of epidemiological data and phylogenetic analysis highlighted a single transmission chain for all cases (Figure 3).

The evolutionary history was inferred using the neighbour-joining method. The evolutionary distances were computed using the Kimura 2-parameter method. Evolutionary analyses were conducted in MEGA6.

### Measles virus genotype B3

Phylogenetic analysis showed that all B3 sequences (n = 27) were identical to the variant Dublin (MVs/Dublin.IRL/8.16; similarity: 100%) which is circulating in European countries and responsible for an ongoing epidemic in Romania [3].

The B3 Dublin variant was first introduced in the east of Milan at the beginning of March 2017, and there was an epidemiological link to the Roma community in France. By the end of March, this variant was detected in a sporadic case returning from Piedmont (northern Italy). Subsequently, the same variant was isolated in a cluster caused by an index case returning from the Apulia Region (southern Italy) and in a cluster that was imported from Romania. In April 2017, genotype B3 was identified in a sporadic case returning from the Lazio Region (central Italy).

During May and June 2017, 12 autochthonous cases were notified in the eastern and south-eastern suburbs of Milan. In June, three nosocomial clusters were identified in an emergency department. Although initially reported as unrelated clusters, the phylogenetic analysis showed a single source of transmission (n = 7, two HCWs, four patients, and one visitor) (Figure 3).

## Conclusions

Eliminating measles and rubella is a core goal of World Health Organization European Region Member States. Effective surveillance is essential for eliminating measles and rubella and its verification [4]. Since the beginning of 2017 and up to 6 August, the Italian Ministry of Health has reported 4,087 cases of measles and three deaths. Most cases occurred in Piedmont and Lombardy (northern Italy), Tuscany, Lazio and Abruzzo (central Italy) and Sicily (southern Italy). Most were older than 15 years (median age: 27 years) and 89% of the cases were not vaccinated. Overall, 42% of the cases were hospitalised and 277 cases were reported among HCWs [5].

Timely measles surveillance is critical to disease control. Identifying and confirming suspected measles cases through surveillance allows early detection of outbreaks and analysis of ongoing transmission in order to mount more effective vaccination measures. MV genotyping can play an important role in tracking transmission pathways during outbreak investigations [6].

From March to June 2017, the genotypes D8 and B3 co-circulated in Milan and surrounding areas. The most common genotype detected was genotype D8, related to 83 sporadic cases and 42 clusters. The high similarity between the D8 MV strains, all belonging to the Osaka lineage, suggests a unique initiating transmission event. This is the first evidence of the Osaka D8 variant in northern Italy, which seems to be replacing the D8 variants that had been circulating in this area since 2013 [7,8].

Moreover, our data show multiple imported cases of B3 MV strains which subsequently spread across Italy and caused several autochthonous cases and clusters. The B3 Dublin variant has replaced the B3 variant Como that had been present in this Italian area from August 2015 to November 2016 [9].

By the end of June 2017, D8 MV strains and B3 MV strains had caused clusters in two major Milanese hospitals. In August (current month), the epidemic is still ongoing and the number of notified cases during the month of July was almost twice the number of cases notified in June. Other authors have already reported that where there is evidence of both nosocomial and community transmission of measles, nosocomial transmission appeared to precede community transmission with a peak of hospital-acquired cases occurring almost two weeks before the peak of the community outbreak [10].

In conclusion, the suboptimal immunisation level (92.5% vaccination coverage rate in Lombardy Region; [11]) and the consequent accumulation of susceptible population have led to an increase in the transmission of measles in northern Italy with detrimental effects on both public health and ongoing measles elimination efforts. Furthermore, the nosocomial outbreaks highlight the importance of improving measles vaccination coverage of healthcare workers.
